# How eye movement and driving performance vary before, during, and after entering a long expressway tunnel: considering the differences of novice and experienced drivers under daytime and nighttime conditions

**DOI:** 10.1186/s40064-016-2148-y

**Published:** 2016-04-27

**Authors:** Yonggang Wang, Longjian Wang, Chen Wang, Yangdong Zhao

**Affiliations:** School of Highway, Chang’an University, P.O. Box 487, Middle Section of South 2 Ring Rd., Xi’an, 710064 Shaanxi China; Hanning Branch, Shaanxi Provincial Expressway Construction Group Co., Dongjiaying, South Zone of Hanzhong Economic and Technological Development Zone, Hanzhong, 723102 Shaanxi China; CCCC First Highway Consultants Co., LTD, 63 Kejierlu Rd., Xi’an, 710075 Shaanxi China

**Keywords:** Tunnel entrance, Novice driver, Visual feature, Driving performance, Transition zone, Safety improvement

## Abstract

**Introduction:**

Driving environment in tunnels is quite different from the ordinary roadway sections, especially the entrance locations, which causes great difficulty in obtaining and interpreting information through fixations and saccades that are relevant to driving safety. Therefore, it is necessary to understand driver's visual behaviors while entering a tunnel so as to take the countermeasures for accident prevention.

**Case description:**

In order to identify the variation of driver’s visual features during the process of tunnel entry, 18 participants were recruited to take part in a driving test conducted in real tunnel sections between Qipanguan toll and Jinshui toll of the G5 expressway in Shaanxi, China. During this test, the drivers’ fixations, saccades and driving performances were captured for further analysis.

**Discussion and evaluation:**

The test data revealed that the driver’s number of fixations, duration of fixations and number of saccades increased gradually at the transition zone. The number of fixations, duration of fixations and number of saccades then drop slightly until the end of the transition zone, and then rise just a little to a stable value after fully adapting to the driving conditions inside the tunnel. Meanwhile, the driver’s number of saccades and saccade amplitude value decreased first, and then increased gradually until reaching a relatively stable value inside the tunnel. Additionally, drivers are more cautious at the transition zone, driving conservatively at lower speed, while decreasing their steering wheel angle and minimizing the vehicle’s lateral deviation. Specially, novice drivers require a longer transition zone before tunnel entry compared to the experienced ones. Moreover, both novice and experienced drivers take more time to get prepared for tunnel entry while driving at night.

**Conclusion:**

Tunnel entrance section is far more dangerous, so drivers should be educated to get ready ahead for tunnel entry, drive cautiously at lower speed and pay full attention to the traffic flow conditions while driving through the tunnel, especially for the novice drivers in night tasks. Tunnel entrance is suggested to have easily identifiable frame design, with effective traffic signs placed at least 170 m before the entrance and gradually changeable LED lighting along the transition zone. All these suggestions provide insight into potential strategies for reducing and preventing traffic accidents and injuries at the tunnel locations.

## Background

Over the past decades, China’s rapidly expanding economy has lead to a dramatic increase in road mileage in mountainous areas, as well as a sharp rise in the number of tunnels. Nowadays, China is the world’s biggest highway tunnel builders. By the end of 2013, there were 12,132 highway tunnels totaling 9,732,400 m in length, including 389 long tunnels between 200 and 18040 m with a total length of 1,246,539 m. As a special kind of infrastructure in highway networks, a large range of differences exists between the luminance inside and outside a tunnel, which can easily cause drivers to experience severe day or night blindness (heliophobia and nyctalopia), also known as a visual “white or black hole effect”. Moreover, the limited interior space and low visibility inside the tunnel are capable of inducing psychological panic. All of these factors contribute significantly to the increased risk of being killed or involved in an accident among tunnel traffic (Ma and Fu 2015).

Drivers need to interpret real time information related to their task in able to judge the current situation, make proper decisions and adjust driving behavior accordingly. While driving, more than 80 % of information, especially the dynamic incentives, is perceived through the driver’s visual movements, which mainly include fixation (eye movement speed <30°/s and acceleration <8000°/s^2^), saccade (eye movement speed >30°/s or acceleration >8000°/s^2^) and blinking. During the normal driving process, drivers obtain nearly all of their information related to driving safety through fixations and saccades. Since a driver’s blinking behavior provides relatively little information compared to fixations and saccades, it is not included in this study. The difference in alignment design and illumination layout is quite significant, which increases the difficulty of the driver’s visual perception and may eventually lead to unsafe driving behaviors or even fatal crashes, especially at tunnel entrance zones (Calvi and D’Amico 2013; Du et al. 2014a).

While the risk of a crash within a tunnel is lower than that on an open road, a crash inside a tunnel often results in much more severe consequences (Amundsen and Ranes 2000). Furthermore, according to the Norway Highway Bureau, about 63.7 % of tunnel accidents occur at entrance zones. Similar results are also found in the analysis of tunnel accidents in China (Ma et al. 2009). These trends suggest that the driver’s difficulty in adapting to the change of light conditions inside and outside of the tunnel is a major factor in tunnel accidents. Therefore, many previous studies have examined drivers’ visual features while driving through a tunnel to assess the risk of crash and identify the potentially hazardous locations related to tunnel travel (Liu et al. 2011; Wang et al. 2015; Yan et al. 2015). Among these studies, illumination inside the tunnel has attracted wide interest (Cheng et al. 2006; Pachamanov and Pachamanova 2008; Bai et al. 2012; Du et al. 2014a; Hu et al. 2014). Du et al. (2014b) reported that the rapid change of illumination at a tunnel’s entrance induces driver’s visual shock or transient blindness that increases the visual load. Bertozzi et al. (2011) also reported similar findings. More specifically, Martens et al. (1997) found that tunnel design (i.e. tunnel wall, lane width, longitudinal profile, road signs and markings, et al.) and illumination have some immediate effects on the driver’s driving behaviors, and similar findings were also reported in the previous researches of Kayser and Oasderski (1991), Kircher and Ahlstrom (2012) and Miller and Boyle (2015). Accordingly, countermeasures should be taken at the transition zones near tunnel entries and exits to decrease the risk of crashing.

Obviously, the complex driving environment at a tunnel entrance brings a great number of information acquisition difficulties to drivers. Since a driver’s visual behaviors are internally associated with his or her information acquisition efficiency, a reasonable understanding of a driver’s visual behaviors is the key to reducing tunnel crashes and improving the overall safety performance of freeway tunnels. For this purpose, eighteen drivers were recruited randomly to attend the driving test in real tunnel sections of the G5 expressway in Shaanxi, China. During the test, drivers’ eye movement (fixations and saccades) and driving performance (speed, steering wheel angle and vehicle lateral deviation) data were captured. Then, the change features concerning the driver’s fixations, saccades and driving performance at both the tunnel entrance and inside of tunnel were analyzed using the test data that have important values for crash and injury prevention at tunnel locations.

## Experiment design

### Participants

Eighteen participants (13 male, 5 female) of 20–40 years old (mean age = 29.4 years, SD = 4.5 years) in good physical and mental health were recruited from the communities in Hanzhong city to participate in the driving test. Among them, 9 participants (7 male, 2 female) have a limited license age of less than 3 years, who are accordingly called the novice group. The remaining 9 participants (6 male, 3 female) belong to the experienced group, and whose average license age was 6.74 years (SD = 2.45).

All participants hold a valid class C1 driver’s license and drive an average of 412 km per week (with a minimum of 207 km and a maximum of 928 km per week). Each participant was required to have either normal or corrected to normal vision and does not take any alcohol, medication or drugs that would impair their driving performance. Additionally, all of the participants were familiar with the local expressway conditions.

### Equipment

The Smart Eye Pro 6.0 three-camera eye tracking system was used to capture the participant’s eye movements, which was mounted on the front windscreen to accurately capture the driver’s eye movements (fixations and saccades) at a frequency of 60 Hz. Two scenes cameras were fixed right above the wheel to record the steering wheel angles and vehicle lateral deviation.

The sensors equipped on the experiment passenger car (BYD G5) were used to collect the vehicle’s speed, acceleration and deceleration as well as other driving behaviors. All recorded data were saved as *txt* files that can be used for offline analysis.

### Driving environment

**Fig. 1:**
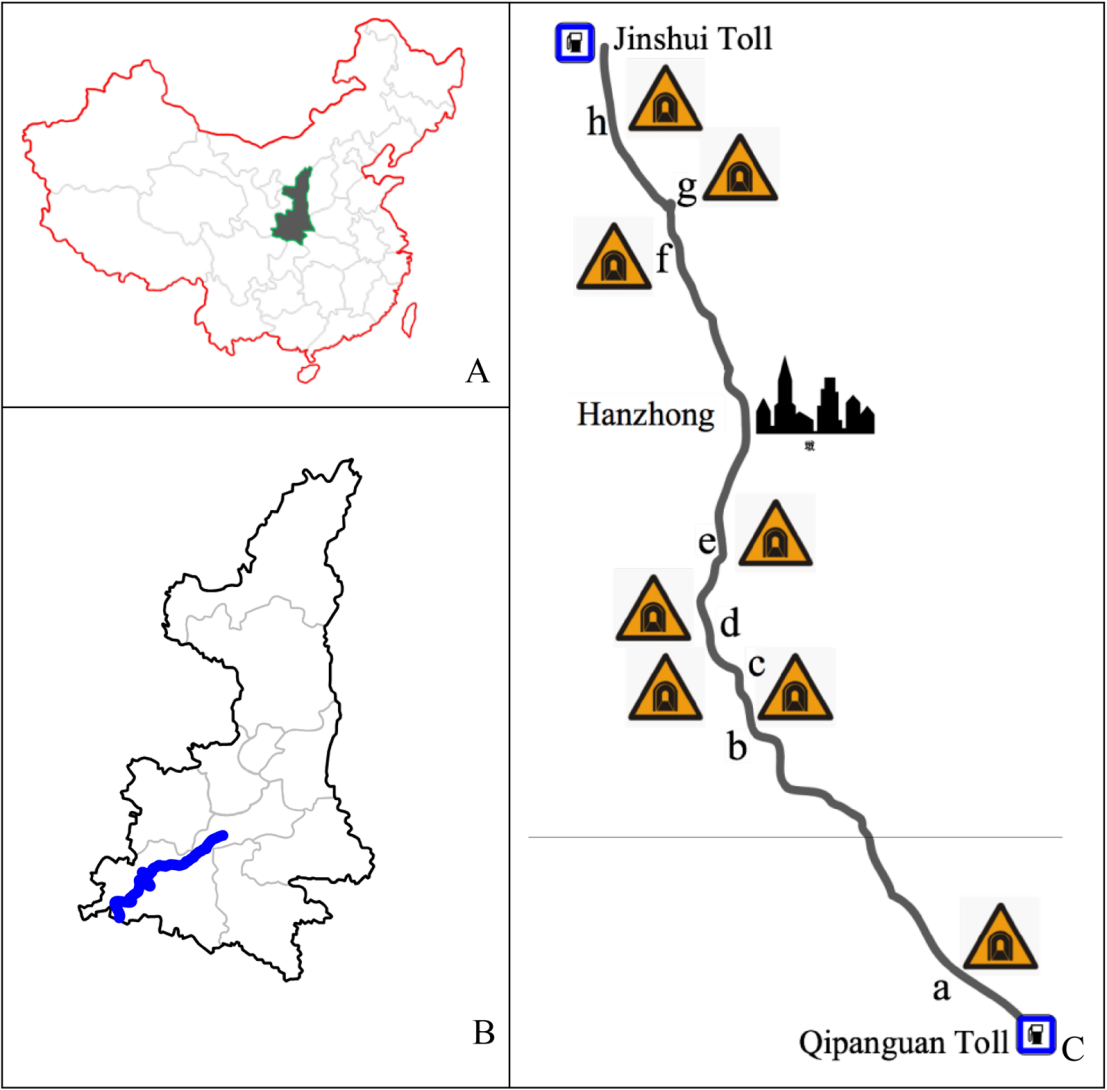
Location of test tunnels. **A** Position of Shaanxi Province in China, **B** Test expressway in Hanzhong, Shaanxi Province, **C** Distribution of tunnels on the test expressway. *Note* *a* Qipanguan Tunnel, *b* Xiejialiang Tunnel, *c* Hejiaxia Tunnel; *d* No. 2 Yuandun Tunnel6, *e* Tielugou Tunnel, *f* No. 2 Youshui Tunnel, *g* No.1 Youshui Tunnel, *h* Qinglongya Tunnel

The Ningqiang–Yangxian Expressway (Fig. 1), a section of the Beijing–Kunming Expressway (G5) in Hanzhong of Shaanxi Province, China, was selected for the driving test. Located in the Qinba Mountains, this section is 198 km long with a posted speed limit of 100 km/h for passenger cars. The cross-section is 15.50 m wide, comprised of a 0.5 m wide grassy median barrier, two 3.50 m wide lanes and a 0.50 m wide shoulder on each side. The alignment for this roadway section consists of 26 tangent–curve configurations. The tangent length varied, ranging between 23 and 1338 m with a mean length of 533 m. The curves range from 48 to 1150 m in radius (mean 328 m), 17 to 197 m in length (mean 69 m) and 6 to 72° in deflection angle (mean 41°). Additionally, the longitudinal grade varies from −3.8 % to +4.4 %.

There are 22 twin bore tunnels in total, as shown in Fig. 1, ranging from 125 to 5335 m (Qipanguan Tunnel) in length. Since the purpose of this study is to examine drivers’ visual behaviors during their visual adaptation from tunnel entrance to the dark environment inside tunnel, this study only considers the tunnels 350 m-long or longer to collect data and analyze the driver’s visual behavior. In total, 8 tunnels and 7 tunnels are included in the driving tests of Qipanguan to Jinshui direction and Jinshui to Qipanguan direction, respectively.

**Fig. 2:**
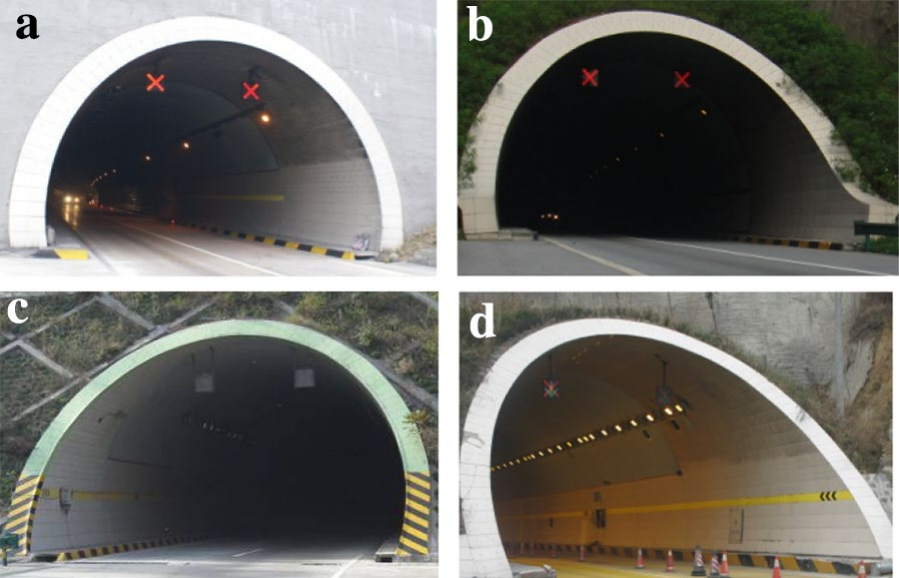
Entrances of typical tunnels along test road. **a** Qinglongya Tunnel, **b** No. 1 Youshui Tunnel, **c** Tielugou Tunnel, **d** Qipanguan Tunnel

The tunnel in each traffic direction consists of two 3.75 m wide lanes and a free height of 5 m. All of the tunnels are equipped with LED lights and traffic signs in advance to alert drivers of tunnel entrances (Fig. 2). Table 1 presents the size and illuminations of each tunnel.

**Table 1:** Tunnel length and illumination conditions

Tunnel	Qipanguan Toll to Jinshui Toll			Jinshui Toll to Qipanguan Toll		
	Location	Length (m)	Number of lighting lamps	Location	Length (m)	Number of lighting lamps
Qipanguan	K1457 + 706	5347	2684	K1457 + 700	5335	2676
Xiejialiang	K1408 + 045	490	202	K1408 + 130	500	206
Hejiaxia	K1399 + 622	444	194	K1399 + 616	460	200
No. 2 Yuandun	K1391 + 997	466	212	K1391 + 842	366	168
Tielugou	K1377 + 352	1401	523	K1377 + 400	1457	523
No. 2 Youshui	K1271 + 440	282	79	K1272 + 056	409	141
No. 1 Youshui	K1270 + 852	798	95	K1271 + 035	708	151
Qinglongya	K1267 + 968	1170	312	K1267 + 945	1352	286

### Data collection

The test was conducted in the middle of May, 2015. Each participant was required to drive along G5 from the Qipanguan Toll to the Jinshui Toll between 2:00 p.m. and 4:30 p.m. and then drive back to the Qipanguan Toll from the Jinshui Toll between 7:30 p.m. and 10:00 p.m. after having supper and a sound rest for performance recovery. In the test, the following hypotheses were given: (1) the driver’s age has no significant effect on visual behaviors; (2) luminance differences in tunnels can be neglected; (3) driver’s visual measures change gradually and no visual shock is considered; (4) variation in the driver’s visual measures reflect psychological and emotional changes.

All of the participants were informed of the purpose, procedure, usage of the eye-tracker and calibration of equipments in the training session before the test. Participants were also allowed to give up at any time if they felt uncomfortable during driving. Participants were asked to give consent about data recording of their visual behaviors in driving before the normal experiment. Then it turned to the formal test session to finish the 2.5 h daytime or night driving task from the Qipanguan Toll or the Jinshui Toll, respectively, with their usual style.

In the following data processing, each participant’s behaviors recorded between 250 m before the tunnel entrance to 250 m inside the tunnel were intercepted and used to analyze the variation of each driver’s visual behaviors. Moreover, these selected sections were divided into three zones: the approaching zone comprised of 250–150 m before tunnel entrance, the transition zone comprised of 150 m before tunnel entrance to 150 m inside the tunnel, and the inside zone comprised of 150–250 m inside the tunnel. 250 m before tunnel entrance was considered the baseline measurement and used to compare driver’s visual change at different zones.

## Results and findings

### Fixations

#### Mean number of fixations

A fixation refers to the period a person maintains his or her visual gaze on a single location, which is a sign of a person’s attention focus. Such a location or area is also called a fixation point, and thus the number of fixations is defined as the total number of fixation points on an area of interest (Swanston and Walley 1984). In driving, once the driver notices a specific region of interest, the number of fixations reflects the number of points of interest that driver is concerned about within the area.

**Fig. 3:**
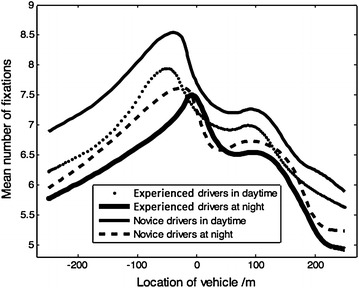
Change of driver’s mean number of fixations

During a visual search task, the number of fixations depends on the amount of driving-related visual information processing, but it is unrelated to the depth of the information (Liu et al. 2011). Figure 3 presents the change in drivers’ mean number of fixations while driving through the tunnel entrance.

A tunnel’s transition zone presents quite a different driving condition, especially for the novice drivers at night. As shown in Fig. 3 and compared with experienced drivers, novice drivers’ average number of fixations per area begins to increase at 50 m (*M* = 7.45, SD = 0.84, *P* < 0.01) and reaches its maximum value at 30 m (*M* = 8.87, SD = 0.75, *P* < 0.05) before tunnel entrance, which indicates that novice drivers have more points of interest while entering a tunnel during the daytime. In the section 50–110 m inside the tunnel, this variable shows a slight increase and then changes much more smoothly.

While performing the night driving task, novice drivers show great interest in spatial information between 170 m (*M* = 6.48, SD = 0.71, *P* < 0.01) and 20 m (*M* = 7.83, SD = 0.57, *P* < 0.05) before tunnel entrance. Their number of fixations fluctuates within a relatively narrow range in the section 30 m (*M* = 6.47, SD = 0.44, *P* < 0.01) to 160 m (*M* = 6.44, SD = 0.51, *P* < 0.01) inside the tunnel. After that, the number of fixations remains steadily.

The number of fixations for the experienced drivers follows a similar pattern of change while driving into a long tunnel, performing a rapid increase before tunnel entrance then decreasing a little to adapt to the human lightning environment inside the tunnel and finally increasing to a stable value. However, the range of change for the experienced drivers is significantly smaller than that of novice drivers.

#### Mean duration of fixations

**Fig. 4:**
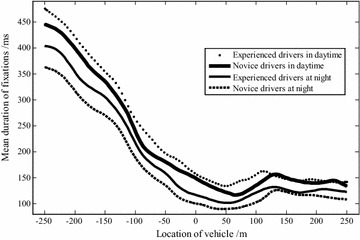
Change of driver’s mean duration of fixations

The duration of fixations (ms) is the total amount of time that a driver fixates on a given area, which reflects the degree of his or her concern on a fixed area and the difficulty of extracting information (She and Chen 2009). As shown in Fig. 4, the average duration of fixations varied significantly in relation to the vehicle’s location.

In the daytime driving period, experienced drivers’ average duration of fixations decreased smoothly by 261.2 % from an average of 475.6 ms (*P* < 0.01) 250 m before tunnel entrance to an average of 131.7 ms (*P* < 0.01) 50 m inside the tunnel, which indicates that these drivers enhanced their focus upon entering the tunnel environment. The average duration of fixations increases gradually soon afterwards to an average of 166.4 ms (*P* < 0.05) at 110 m inside the tunnel, then decreases to an average of 143.4 ms (*P* < 0.01) at 170 m, and finally remains constant at an average of 140 ms (*P* < 0.05). During the night driving period, the average duration of fixations at all three tunnel entrance zones is lower than that during the daytime, but it follows a similar decrease–increase–decrease pattern.

As for the novice drivers, as shown in Fig. 4, the average duration of fixations decreases dramatically while driving between 250 m out of tunnel and 70 m inside the tunnel. After entering the tunnel, the duration of fixations increases just a little and then quickly decreases back to the steady-state value. All of these observations indicate that the novice drivers are more interested in the tunnel environment and try to detect more necessary information for safe driving.

#### Percentage of time with eyes off-road

The proportion of time spent with eyes off the road (%) indicates whether and how a driver’s current focus of visual attention is aroused (Xian and Jin 2015).

In view of spatial distribution of driver’s fixation points, repeated ANOVA analysis found that this variable differs significantly in relation to the location of the vehicle as compared to the baseline for both experienced (approaching zone: *M* = 18.63 %, SD = 3.47 %, *P* < 0.01; transition zone: *M* = 11.54 %, SD = 1.62 %, *P* < 0.01; inside zone: *M* = 14.28 %, SD = 2.36 %, *P* < 0.01) and novice drivers (approaching zone: *M* = 16.53 %, SD = 2.27 %, *P* < 0.01; transition zone: *M* = 9.17 %, SD = 1.24 %, *P* < 0.01; inside zone: *M* = 13.43 %, SD = 1.98 %, *P* < 0.01) under night driving conditions.

Differences in the daytime driving conditions are also significant, all *P* < 0.05, but the mean proportion of time spent with eyes off-road is less affected by tunnel environments.

#### Maximum off-road fixation time

A driver will be more likely to be involved in a crash if his/her fixation remains away from the road for 2 s or more (Rockwell 1988). Accordingly, the maximum off-road fixation time (s) can be used to determine whether a driver is distracted and assess the potential risk under specific driving conditions.

**Fig. 5:**
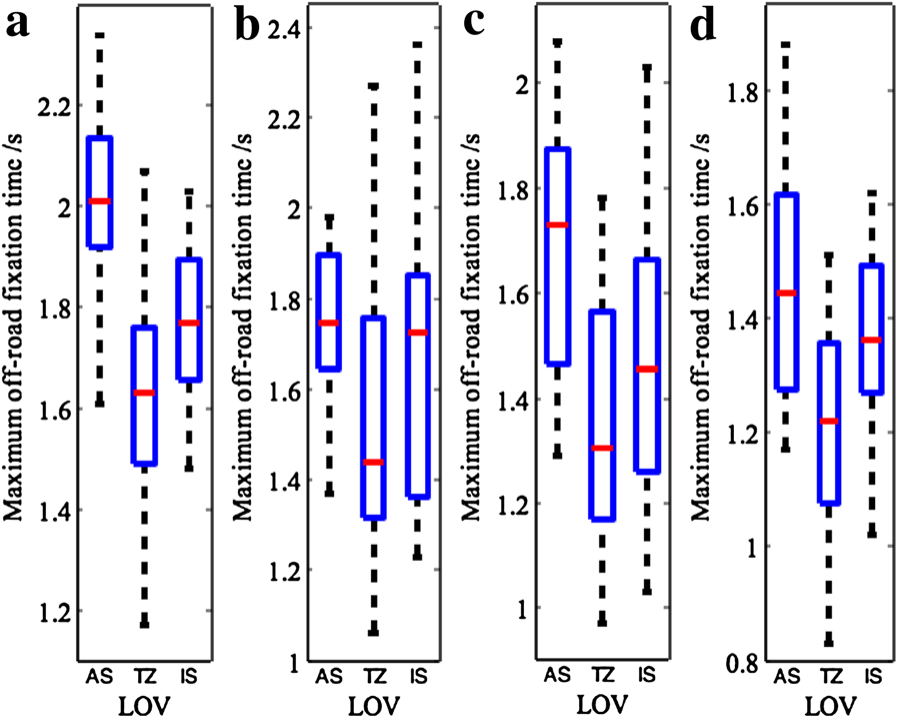
Change of driver’s maximum off-road fixation time. **a** Experienced drivers in daytime, **b** experienced drivers at night, **c** novice drivers in daytime, **d** novice drivers at night

 Figure 5 shows that the maximum off-road fixation time varies significantly with particular vehicle locations and driver groups. While approaching the tunnel entrance, drivers need to interpret the necessary information (i.e. roadside signs, overall size of tunnel entrance, possible risk, etc.), which easily distracts their attention. Thus, the maximum off-road fixation time is also relatively longer (the experienced in daytime: *M* = 2.01 s, SD = 0.18 s, *P* < 0.05; the experienced at night: *M* = 1.74 s, SD = 0.18 s, *P* < 0.01; the novice in daytime: *M* = 1.69 s, SD = 0.24 s, *P* < 0.05; the novice at night: *M* = 1.46 s, SD = 0.21 s, *P* < 0.01), compared to those outside and inside the tunnels.

On the other hand, this indicator is significantly shorter across the transition zone (the experienced in daytime: *M* = 1.61 s, SD = 0.22 s; the experienced at night: *M* = 1.53 s, SD = 0.32 s; the novice in daytime: *M* = 1.36 s, SD = 0.24 s; the novice at night: *M* = 1.21 s, SD = 0.19 s), because nearly all of the driver’s attention is focused on the wheel as well as on vehicles ahead of the driver or in adjacent lanes, and so they are not likely to be distracted. All *P* values are found to be no more than 0.05.

#### Standard deviation of visual search angle

The horizontal and vertical visual search angles (°) characterize a person’s visual search breadth from the average fixation point, meaning that any change in value indicates the influence of attention by other stimuli (Kim and Cave 1995).

**Fig. 6:**
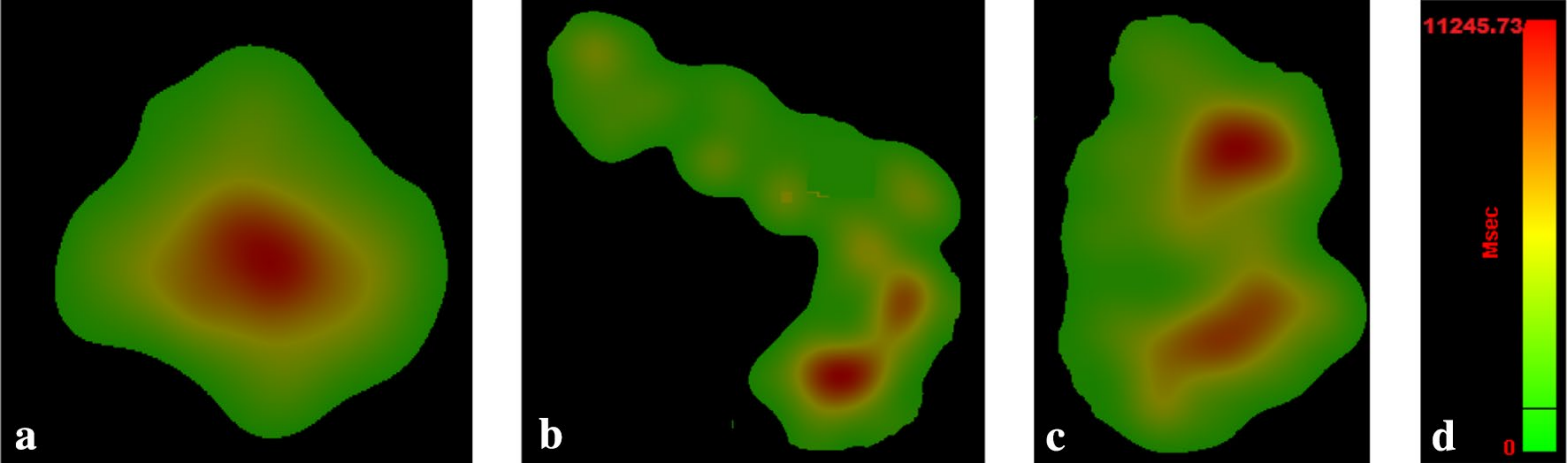
Change of novice drivers’ visual search area at night. **a** Approaching zone, **b** transition zone, **c** inside zone, **d** scale ratio

In the test, significant differences were found in the standard deviation of horizontal and vertical search angles while entering a tunnel (*P* < 0.05). Figure 6 presents the average change of the visual search area for novice drivers during the night driving task.

In the approaching zone, novice drivers focus their full attention on the tunnel entrance ahead, which makes their fixation points limited to the area around the tunnel entrance, as shown in Fig. 6a. While driving across the transition zone, novice drivers needed to evaluate the traffic states in their current and adjacent lanes, and therefore their corresponding visual search area is psychologically quite restricted in a V-type region, as shown by Fig. 6b, especially on the right. In the tunnel, drivers paid the most attention to traffic flow, so their visual search area is focused on the current and adjacent lanes (Fig. 6c).

### Saccades

#### Mean number of saccades

The mean number of saccades represents the frequency of information search, which decreases as the complexity of a cognitive task increases (Liu et al. 2011).

**Fig. 7:**
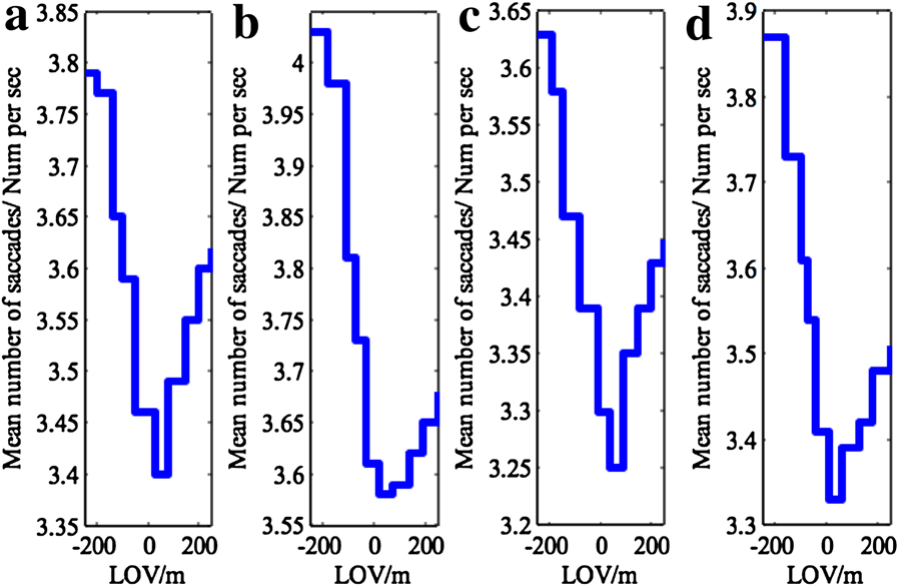
Change of driver’s mean number of saccades. **a** Experienced drivers in daytime, **b** experienced drivers at night, **c** novice drivers in daytime, **d** novice drivers at night

Test data analysis shows that the mean number of saccades reduces from 3.79 times per second at 250 m ahead of the tunnel entrance to 3.40 times per second at 30 m inside the tunnel for experienced drivers in the daytime. Then, the mean number of saccades increases a little to a stable value of 3.62 times per seconds after full adaptation to the new environment inside the tunnel (Fig. 7a). While performing the night task, their mean value decreased from 4.03 times per second at 250 m ahead of the tunnel entrance to 3.58 times per second at 30 m inside the tunnel, and then bounced back up to 3.68 times per second, where it stabilized (Fig. 7b).

The novice drivers’ mean number of saccades also sharply decreases until reaching the minimum value of 3.25 times per second at 40 m and 3.33 times per second at 40 m inside the tunnel, and then rises lightly to 3.45 times and 3.51 times per second, where the value stabilizes in daytime and night tasks (Fig. 7c, d), respectively.

#### Mean saccade amplitude

The saccade amplitude variable (°) shows the target position of a given driver’s visual search and indicates the eye movement velocity from one fixation point to another (Yan et al. 2015), which is significantly affected by traffic flow and the roadside environment, and can be measured by the axis or angle of eye rotation.

**Fig. 8:**
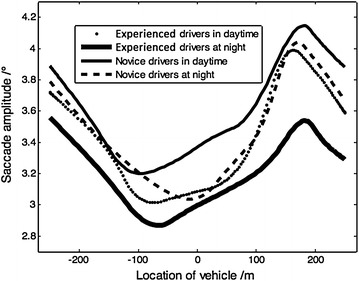
Change of driver’s mean saccade amplitude

Through statistics collected from the saccade behaviors of these 18 drivers, the saccade amplitude was found to be affected by environmental conditions (all *P* < 0.05), as shown in Fig. 8. While entering the tunnel, drivers’ attention focused on entrance information, and then they tried to search and adapt to the new circumstances inside the tunnel, during which driver’s saccade amplitude decreases sharply in the transition zone and then increases slightly once inside the tunnel. As soon as drivers acclimate to the inside conditions, the variable decreases to a stable value.

Of course, experienced drivers tend to adapt more rapidly to the changing environmental conditions inside the tunnel, and their values of saccade amplitude are thus relatively smaller. According to the results, it is more difficult for both experienced and novice drivers to interpret safe driving information related during the night driving task, and so as expected their saccade amplitudes are lower.

#### Mean saccade speed

Mean saccade speed (°/s) is the average ratio of each saccade angle to its duration. Mean saccade speed correlates with decision making and impulsivity in previous fixation and visual search efficiency.

As shown in Table 2, saccade speed also varies according to different locations and driver groups (*P* < 0.05 in all cases). Novice drivers have a higher saccade speed average than experienced drivers, which reflects their information seeking interest while entering tunnels. Additionally, both experienced and novice drivers have the lowest mean and maximum values of saccade speed while driving through the approaching zone at night.

**Table 2:** Saccades speed of experienced and novice drivers in daytime and night driving

Drivers	Location	Daytime driving				Night driving			
		M (°/s)	SD (°/s)	*P*	Max (°/s)	M (°/s)	SD (°/s)	*P*	Max (°/s)
Experienced	AS	22.39	4.56	<0.01	238.76	20.16	3.71	<0.01	257.64
	TZ	46.37	3.23	0.016	412.33	38.77	4.93	0.034	456.17
	IS	31.69	5.47	<0.01	317.46	28.39	3.39	0.020	336.42
Novice	AS	29.16	3.68	0.023	303.20	27.46	5.69	<0.01	335.08
	TZ	56.51	6.76	<0.01	491.46	49.33	4.74	<0.01	521.77
	IS	40.65	4.55	<0.01	411.63	34.65	6.17	0.017	448.61

### Driving performance

#### Mean speed

For the experienced drivers, the observed mean speed value (m/s) is significantly lower within the transition zone (daytime driving: *M* = 18.37 m/s, SD = 2.61 m/s, *P* < 0.05; night driving: *M* = 17.08 m/s, SD = 1.63 m/s, *P* < 0.01), but after entering the tunnel, it increases slightly (daytime driving: *M* = 19.39 m/s, SD = 1.15 m/s, *P* < 0.01; night driving: *M* = 18.58 m/s, SD = 2.29 m/s, *P* < 0.05).

Due to less driving experience, the novice drivers (approaching zone: *M* = 19.27 m/s, SD = 2.06 m/s, *P* < 0.05; transition zone: *M* = 16.39 m/s, SD = 1.88 m/s, *P* < 0.01; inside zone: *M* = 17.84 m/s, SD = 1.71 m/s, *P* < 0.01) can drive carefully with a relatively lower mean speed during daytime driving, and they drive even more conservatively and at a lower speed while performing night driving tasks.

#### Standard deviation of steering wheel angle

Steering angle variation (°) is proposed to determine the corrections that should be applied to the steering system to maintain the proper angle. Here, no significant difference for this indicator was found between experienced drivers in daytime and night driving conditions.

For novice drivers, however, location and driving time show a significant effect (approaching zone in daytime: *M* = 1.94°, SD = 0.51°; approaching zone in night: *M* = 1.75°, SD = 0.47°; transition zone in daytime: *M* = 1.69°, SD = 0.51°; transition zone in night: *M* = 1.61°, SD = 0.64°; inside zone in daytime: *M* = 1.68°, SD = 0.39°; inside zone in night: *M* = 1.72°, SD = 0.51°) concerning the standard deviation of steering wheel angle (all *P* < 0.05).

#### Vehicle lateral deviation

The lateral deviation indicator represents a driver’s ability to maintain a central, steady and safe lane position while driving (Gemou 2013). Root-mean-squared error of the lateral distance between a vehicle’s center and the lane center (m) is captured and used to determine a vehicle’s average lateral deviation while entering a tunnel.

Analysis results show that both experienced and novice drivers can keep a small lateral deviation (0.2 m or less) inside tunnels and while performing either daytime or night driving tasks. Within the transition zone, all drivers maintained a lateral deviation of less than 0.3 m during the daytime period; however, that of novice drivers in night driving conditions expanded to as large as 0.45 m. Experienced drivers under night driving conditions and within the approaching zone kept a stable lateral deviation of no more than 0.4 m, whereas novice drivers kept a wider lateral deviation ranging from 0.3 m to 0.5 m.

## Conclusions and discussions

Tunnels provide quite a different environment for drivers, especially at the entrance location, which causes great difficulty for drivers to interpret information from roadway and traffic conditions through fixations and saccades. Using data from an on-the-road driving test including 18 drivers on the Qipanguan–Jinshui section of the G5 Expressway, this research has explored the variation characteristics of driver’s fixation, saccade and driving performance while approaching driving through tunnel entrances.

From the statistical results, it can be found that a driver’s number of fixations, duration of fixations and number of saccades begin to increase gradually from the transition zone in order to interpret more information and then decline after a gradual adaptation to the new driving environment inside the tunnel. On the contrary, the drivers’ saccade amplitude decreases greatly both within the transition zone and on the road approaching this zone, and then increases to a stable value inside the tunnel. Meanwhile, the drivers’ visual search area focuses on the tunnel entrance straight ahead, but it expands its searching angle with more attention on new driving conditions once inside the tunnel, especially from the right hand side. The corresponding fixation point angle expands from middle points to around ones. Additionally, drivers are more careful and cautious, driving at lower speeds, and resulting in a smaller steering wheel angle and lateral deviation value while driving across the transition zone. This is especially the case for novice drivers at night.

Our research results also indicate that novice drivers require a longer transition period (170 m before tunnel entrance and 50 m inside the tunnel) to get prepared for tunnel entry while performing night driving tasks. The change of their visual indicators, such as the number of fixations, duration of fixations, number of saccades and saccade speed, etc., is more significant than that of experienced drivers. They require more time to react and make decisions for tunnel entrance, which results in them reducing their driving speed and thus driving more cautiously during this period.

These findings imply some considerations for tunnel design and safety management measures. First, a tunnel entrance should be set in a straight-line segment instead of a curve, and the frame of the tunnel entrance should be easily recognizable to drivers so that they will have enough time to understand the current status of the tunnel entrance and prepare for an effective safe driving in the tunnel. Second, the frame design and facilities layout should be different from the background rock mass and plants, which will also help drivers identify the existence of the tunnel entrance. Third, traffic signs should be placed at least 170 m before a tunnel entrance to address the needs of novice drivers at night, which complies with the previous findings (Narisada and Yoshikawa 1974; Verwey 1995). Fourth and finally, the first 50 m section after entering a tunnel should be equipped with changeable LED lighting, providing a gradual change from the natural bright or dark lighting outside the tunnel to a quite different lighting environment inside the tunnel, ensuring the driver’s dynamic dark adaptation. Additionally, drivers should be educated to comply with traffic sign warnings and reduce their traveling speed consciously while driving through tunnels.

Due to limited time and funds, the sample size of this study was small and may not be representative of all Chinese drivers; this methodological flaw means that the results may be not statistically sound. Moreover, driver’s visual indicators are highly sensitive to individual physiological and psychological conditions (Das et al. 2015), which can be dramatically affected by ambient stimulus, and consequently, the collected visual data may potentially contain inaccuracies due to temporary environmental effects.

Future studies would systematically focus on how to capture driver’s visual data through reliable and accurate test techniques and thus more precisely identify the potential risk of driving though tunnels. Furthermore, it is important to link these eye movement metrics to actual driving behaviors, such as lane change, acceleration and deceleration, and vehicle following, as well as bad weather conditions, etc., so as to examine how the tunnel environment affects driver’s visual driving behaviors over a period of time while also examining how personality conjointly influences this relationship. Lastly, driver’s performance recovery techniques also merit further research.
